# Synthesis of Nanocrystalline PuO_2_ by Hydrothermal and Thermal Decomposition of Pu(IV) Oxalate: A Comparative Study

**DOI:** 10.3390/nano13020340

**Published:** 2023-01-13

**Authors:** Viktoria Baumann, Karin Popa, Olaf Walter, Murielle Rivenet, Gérald Senentz, Bertrand Morel, Rudy J.M. Konings

**Affiliations:** 1 Univ. Lille, CNRS, Centrale Lille, Univ. Artois, UMR 8181—UCCS—Unité de Catalyse et Chimie du Solide, F-59000 Lille, France; 2 European Commission, Joint Research Centre, 76344 Karlsruhe, Germany; 3 ORANO, 92320 Chatillon, France

**Keywords:** nanopowder, plutonium dioxide, wet chemistry route

## Abstract

In recent years, the hydrothermal conversion of actinide (IV) oxalates into nanometric actinide dioxides (*An*O_2_) has begun to be investigated as an alternative to the widely implemented thermal decomposition method. We present here a comparison between the hydrothermal and the conventional thermal decomposition of Pu(IV) oxalate in terms of particle size, morphology and residual carbon content. A parametric study was carried out in order to define the temperature and time applied in the hydrothermal conversion of tetravalent Pu-oxalate into PuO_2_ and to optimize the reaction conditions.

## 1. Introduction

Nanotechnology is thought to offer applications that are of interest in the nuclear field [1]. In nuclear medicine, tumor-target nanoparticles of radionuclides are used for cancer radiation therapy without harming healthy tissue [2]. In nuclear facilities, nanotechnology is relevant in the fabrication of nuclear fuel, in separation processes and in waste disposal, as it improves the efficiency of these processes [1]. In order to study the advantages of nanotechnology in more detail, the versatile and effective synthesis of *An*O_2_ nanoparticles is highly desirable. The conventional thermal conversion of Pu(IV) oxalate to PuO_2_ not only occurs at high temperatures, but also results in platelet-shaped particles, even in the mild variant at 600 °C [3]. In addition to solvothermal methods [4,5,6,7,8], which require organic solvents, or radiolysis [9], hydrothermal synthesis [10,11] has gained importance in recent years as it reduces both temperature and particle size. While the studies by Tabata et al. and Shirasaki et al. exploited the property of water as a polar solvent at the critical point, albeit with the requirement of temperatures above 400 °C [12,13,14], Walter et al. proposed the mild hydrothermal decomposition of actinide (IV) oxalates (*An* = Th, U, Pu) under hot compressed water at temperatures below 250 °C, yielding nanocrystalline *An*O_2_ [15]. Popa et al. extended the method to other actinides as well as their associate solid solutions [16,17].

The nanoparticles obtained are quasi-spherical and typically below 10 nm in diameter. While the thermal process leads to two-dimensional aggregates of nanoparticles that complicate the sintering process, the hydrothermal process leads to soft agglomerates of nanocrystals that no longer exhibit the pre-organized morphology of the original oxalate and are held together by surface interactions. The sintering behavior of such powders is excellent and allows the process temperature to be decreased, as shown in uranium dioxide, thorium dioxide and their solid solutions [17,18,19]. The recent approach is thus to synthesize nanometric PuO_2_ and take advantage of the benefits associated with this low-temperature method.

The morphology of the *An*O_2_ powders resulting from the hydrothermal decomposition of actinide oxalates can be controlled by experimental parameters such as the pH value, temperature or time during the hydrothermal conversion. Manaud et al. showed that a higher process temperature (220–250 °C) is advantageous for the hydrothermal conversion of U(IV) oxalate into uranium (IV) dioxide as the oxide stoichiometry decreases and leads to a lower proportion of U_4_O_9_ in the mixture of UO_2+x_/U_4_O_9_ [20]. The initial pH value can also determine the shape and size of the obtained particles [21]. To the best of our knowledge, no parametric study has been conducted on PuO_2_ case in order to define and optimize the reaction conditions.

On the other hand, the carbon content is an important parameter and maximal values are defined for nuclear fuels [22]. Together with the specific surface area, the carbon content is one of the crucial factors in the determination of the quality of the powders used for fuel production [23].

Herein, we present a direct comparison between the hydrothermal and the conventional thermal conversion of Pu(C_2_O_4_)_2_·6H_2_O into PuO_2_ in terms of the particle size, shape of agglomerates/aggregates and carbon content. Furthermore, we report a systematic investigation of the effect of the temperature and reaction time on the plutonium-oxalate hydrothermal-decomposition process. The nanocrystalline powders were investigated by X-ray diffraction (XRD) and scanning electron microscopy (SEM).

## 2. Materials and Methods

### 2.1. Synthesis

Plutonium is a radioactive element. Only certified and licensed laboratories are allowed to work with it and the safety and radioprotection regulations must be respected. The work described here was performed at the JRC Karlsruhe accordingly.

The synthesis of *An*(IV)-oxalates and their decomposition under hydrothermal conditions have already been described in the literature [15,16,17,18]. Accordingly, a stock solution of Pu(IV) was prepared by dissolving bulk plutonium dioxide in concentrated nitric acid. After dilution with 0.5 M HNO_3_, the plutonium concentration was nearly 0.8 M in 4 M HNO_3_. The oxalic acid dihydrate was supplied by Merck and was of analytical grade. The Pu(C_2_O_4_)_2_·6H_2_O was prepared by direct precipitation with a 0.5-mole-per-liter oxalic-acid solution in slight excess under continuous stirring. A precipitate of a champagne-brown color formed according to the following reaction Equation (1):

Pu^4+^ + 2C_2_O_4_H_2_ + 10H_2_O → Pu(C_2_O_4_)_2_·6H_2_O + 4H_3_O^+^ (1)

The Pu(IV) oxalate precipitate was separated from the reaction solution by decantation. After separation from the acidic media, it was washed with distilled water, ethanol and acetone to remove any trace of free oxalic acid or traces of nitric acid and to decrease the polarity of the sample. The hydrothermal conversion of Pu(C_2_O_4_)_2_·6H_2_O into PuO_2_ was carried out in a Teflon-lined steel autoclave made for hydrothermal synthesis and Teflon insets with a free volume of 12 mL. The following General Reaction (2) occurred:

Pu(C_2_O_4_)_2_·6H_2_O → PuO_2_ + 2CO + 2CO_2_ + 6H_2_O (2)

In this regard, amounts of 100 mg (0.19 mmol) of Pu(C_2_O_4_)_2_·6H_2_O were placed into a Teflon inset and covered with 5 mL of distilled water. The autoclave was screwed tightly, placed in a heating jacket and heated to the desired temperature by built-in heating patrons. During the decomposition, gaseous CO/CO_2_ and water vapor formed, which increased the pressure inside the autoclave (autogenic pressurization).

The duration of the decomposition indicated in our work was the time that elapsed from reaching the desired temperature to the end of heating process. The autoclave was cooled to room temperature by convection and this interval was not included in the reaction time. The cooling time was between 2 and 5 h, depending on the process temperature. All samples obtained in this way were separated from solution, washed with distilled water, ethanol and acetone and dried in air.

For the parametric study, the temperature’s influence was examined by varying the temperature between 100 °C and 280 °C, while the reaction time was set to 4 h. The influence of the reaction time on the decomposition of Pu(IV) oxalate was studied at different reaction times between 1 and 24 h at a given temperature fixed at 220 °C.

As comparison material for thermal decomposition, plutonium oxalate was calcined in a furnace at 700 °C for 6 h under argon flow (200 °C/h heating and cooling rates).

The obtained products were compared in terms of their properties, focusing on the crystallite size, the morphology and the residual carbon content, as it is assumed that these properties strongly influence the sintering behavior.

In order to investigate the influence of the metal-oxidation state on the plutonium oxalate during hydrothermal decomposition, Pu(III) oxalate was synthesized by reducing Pu(IV) (0.07 M in 2 M HNO_3_) in Pu(III) with 2-mole-per-liter hydroxylamine-nitrate solution, followed by oxalate precipitation with 0.5-mole-per-liter oxalic-acid solution. Both the Pu(III) solution and Pu_2_(C_2_O_4_)_3_·10H_2_O presented a characteristic blue color, which was sufficient evidence of successful reduction [24]. The precipitate was separated, washed with distilled water, ethanol and acetone and air-dried overnight.

### 2.2. Physicochemical Characterization

#### 2.2.1. X-ray Diffraction (XRD) Characterization and Evaluation of the Crystallite Size

All samples were analyzed by powder X-ray diffraction. Room temperature XRD patterns were collected on a Bruker D8 diffractometer mounted in a Bragg–Brentano configuration with a curved Ge (1,1,1) monochromator and a ceramic copper tube (40 kV, 40 mA) and supplied with a LinxEye position sensitive detector. The crystallite-size calculation was based on the half-width of six selected peaks in the 2θ range between 45° and 80° using HighScore Plus software (version: 3.0.4) for profile fitting. The obtained full width at half maximum (FWHM) was converted by Scherrer’s Equation (3) into the crystallite size *D*:

(3) $$\;D=\frac{K\lambda }{\beta_{hkl}\cos\theta }$$

where *K* = 0.94 for spherical crystals with cubic symmetry and *ß_hkl_* the measured FWHM. The calculated crystallite size was therefore an average value of the six selected peaks.

#### 2.2.2. Microscopic Characterization

Scanning electron microscope (SEM) analyses were performed using a Philips XL40 SEM with an acceleration voltage of 25 kV. To reduce charging, the samples were placed on the carbon sticker and coated with carbon.

Transmission electron microscope (TEM) analyses were performed on a TecnaiG2 (FEI™) 200-kilovolt microscope equipped with a field emission gun, modified during its construction to enable the examination of radioactive samples. The samples for the TEM investigations were obtained by dropping suspended samples on a TEM grid and evaporating the solvent.

#### 2.2.3. Carbon Analysis

The amount of residual carbon was analyzed using carbon/sulfur CS-800 Double Dual Range by ELTRA. The samples were placed in a ceramic crucible, to which an accelerator such as iron or tungsten was added and melted in a pure oxygen atmosphere. The carbon content was determined with infrared cells.

## 3. Results and Discussion

### 3.1. Effect of Temperature

**Figure 1:**
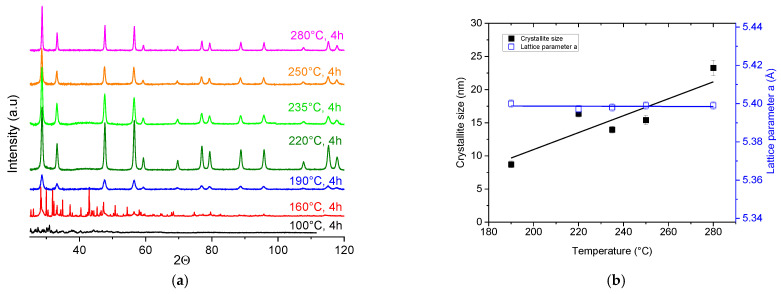
(**a**) Evolution of XRD patterns obtained for samples by hydrothermal decomposition of Pu(C_2_O_4_)_2_·6H_2_O in the temperature range from 100 °C to 280 °C for 4 h. Note that under the selected conditions, PuO_2_ forms in pure phase above 190 °C; (**b**) variation in the crystallite size and lattice parameter *a* of PuO_2_ as a function of temperature.

The temperature’s influence was examined by performing experiments at 100 °C, 160 °C, 190 °C, 220 °C, 235 °C, 250 °C and 280 °C at a reaction time fixed at 4 h. Nanocrystalline PuO_2_ was formed under these conditions via hydrothermal conversion for temperatures above 190 °C. Crystallization took place in the space group *Fm-3m* (225) with a fluorite-type cubic structure, as shown in Figure 1a. The crystalline structures of the samples prepared at lower temperatures were not fully resolved and belonged to reaction intermediates that were stable under the given reaction conditions.

The literature proposes several reaction mechanisms for the thermal decomposition of plutonium oxalate and corresponding intermediates have been identified, such as water-free Pu^IV^(C_2_O_4_)_2_, Pu(III) hydrated oxalates or phases with oxalate-carbonate structures and Pu^IV^OCO_3_ [23,25,26,27].

In our study, the Pu(III) oxalate was hydrothermally converted to PuO_2_ by heating at 160 °C for 2 h while the Pu(IV) oxalate was fully converted after 4 h of hydrothermal treatment at 190 °C. The treatment of the Pu(IV) oxalate at 160 °C for 4 h resulted in an incomplete reaction, which led to nanometric PuO_2_ accompanied with a secondary, unidentified phase, which was present in non-negligible amounts.

The crystallite sizes of the obtained PuO_2_ samples were assessed based on the XRD data and are shown in Figure 1b as a function of temperature. With increasing temperature, the crystallites’ size increased from 8.7 ± 0.5 nm up to 24.8 ± 0.5 nm. This was larger than the 3.6 nm at 95 °C previously reported by Walter et al. [15], due to the lower reaction temperature (but longer reaction time). The average lattice parameter *a* was 5.398(1) Å, corresponding to the PuO_2_ of oxygen stoichiometry, and remained constant regardless of the reaction conditions.

**Figure 2:**
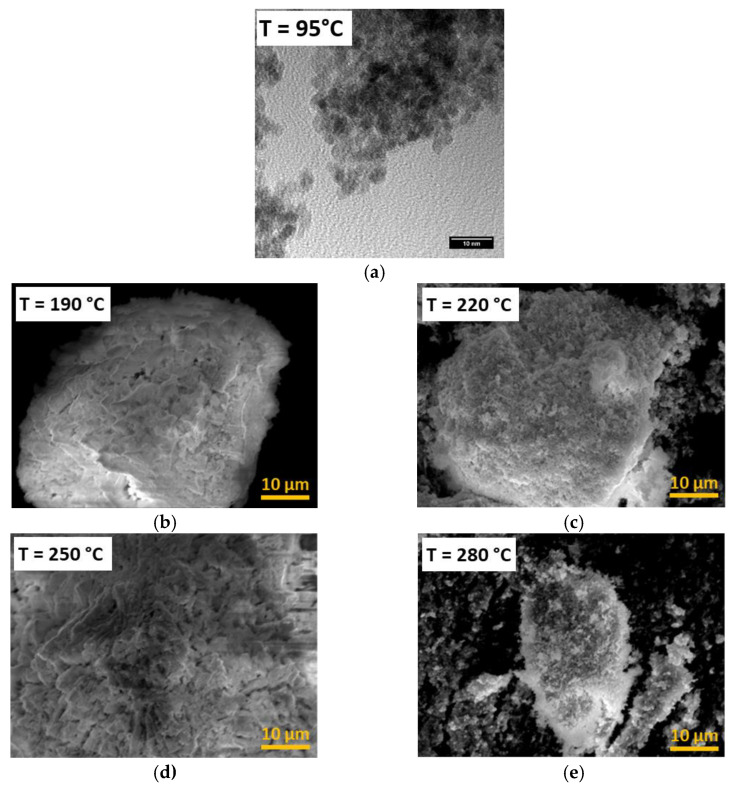
(**a**) Transmission-electron picture of PuO_2_ obtained by using the hydrothermal method at 95 °C; (**b**–**e**) scanning-electron micrographs of PuO_2_ nanoparticles prepared by hydrothermal decomposition of Pu(IV) oxalate at different temperatures.

The sizes of the crystallites observed in the TEM were in very good agreement with the results extrapolated out of the powder XRD, as shown in Figure 2a, for a nanopowder obtained at 95 °C in a previous study [15]. This seminal report [15] presented an extended discussion on the correlation of the particle size obtained by the two characterization methods (XRD and TEM); thus, we do not include it here. Unfortunately, due to technical constraints, the performance of systematic TEM observations during the present study was not possible. However, we used the SEM to examine the behavior of the agglomerates, which were assemblies of several individual crystallites, although the morphology of the powder was too small to be determined by SEM with 2000× magnification (Figure 2). Nevertheless, it can be seen that the plate-shaped morphology of the plutonium oxalate disappeared and that the structures of the samples were porous. All the samples produced at temperatures above 190 °C showed similar morphologies.

### 3.2. Effects of Duration

**Figure 3:**
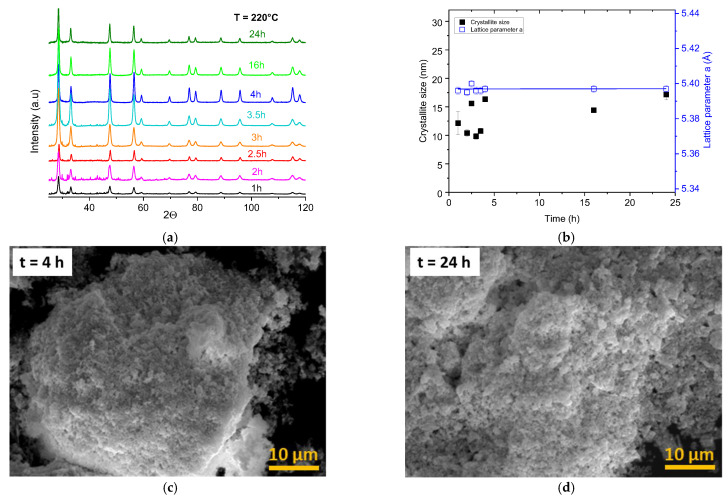
(**a**) Evolution of XRD patterns obtained for samples after hydrothermal conversion of Pu(C_2_O_4_)_2_·6H_2_O at the temperature of 220 °C for different durations; (**b**) variation in the crystallite sizes and lattice parameter with the reaction time (**c**,**d**); scanning-electron micrographs of PuO_2_ nanoparticles prepared after hydrothermal treatment for 4 and 24 h.

The influence of the reaction time was examined by performing the hydrothermal decomposition of the Pu(IV) oxalate for a given temperature of 220 °C by performing experiments of 1 h, 2 h, 2.5 h, 3 h, 3.5 h, 4 h, 16 h and 24 h, respectively. As can be seen from XRD data shown in Figure 3, the hydrothermal decomposition of the plutonium oxalate hydrate resulted in PuO_2_ nanocrystals with cubic structures of the fluorite type in all cases. In addition to the PuO_2_ phase, additional peaks occurred for shorter processing durations (less than 3.5 h). This is likely to have been due to the incomplete decomposition of the plutonium oxalate.

The crystallites’ size calculated by using the XRD data were between 10.8 ± 1 nm and 17.2 ± 1 nm (Figure 3a) for reaction times of 3.5 h and 24 h, respectively. Higher values were determined for longer duration times, but it can be clearly seen that the effect of the reaction time on the particle growth was weaker compared to the effect of temperature shown above. The average lattice parameter *a* was 5.398(1) Å and, as in the previous study, independent of the reaction conditions. The morphologies of the samples were not affected by the reaction time, with each of the samples showing a porous morphology with no evidence of a two-dimensional-preorganized structure.

### 3.3. Hydrothermal vs. Thermal Decomposition

**Figure 4:**
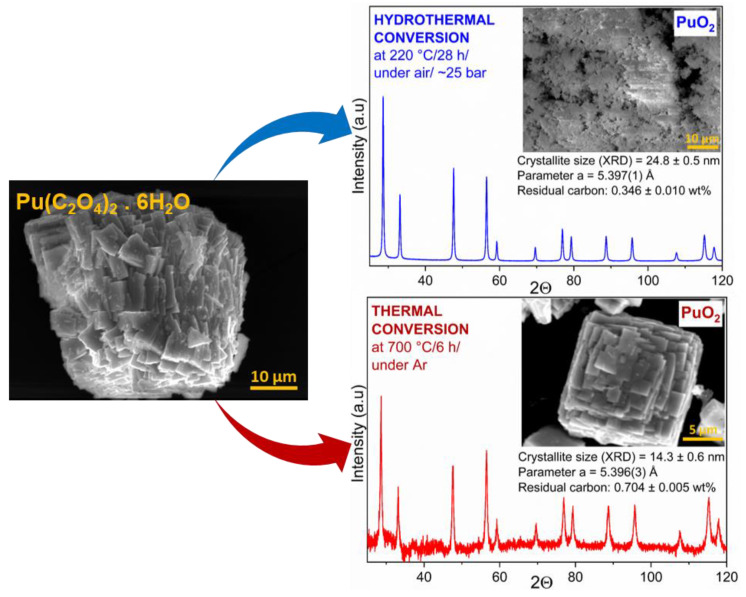
Scanning-electron micrographs of Pu(C_2_O_4_)_2_·6H_2_O (**left**) and PuO_2_ samples prepared by hydrothermal (**top**) and thermal conversion (**bottom**) of Pu(IV) oxalate, together with the corresponding XRD patterns, crystal properties and residual carbon content.

The comparison between the plutonium-dioxide powders from the hydrothermal and the conventional thermal decomposition of the Pu(IV) oxalate is shown in Figure 4. It was demonstrated that Pu(IV) oxalate has a plate-like morphology, which is typical of actinide-based oxalates [3,28]. This pseudomorphic appearance of the particles was retained after the thermal decomposition to PuO_2_ (700 °C/6 h/under Ar) and led to the formation of aggregates of micrometric sizes. By contrast, the hydrothermal method (220 °C/ 28 h/25 bar under air) resulted in the formation of soft agglomerates, which was most likely due to the free mobility of the material in the solution. The recorded XRD pattern gave PuO_2_ (fluorite structure, cubic *Fm-3m* (225)-space group) with a lattice parameter *a* of 5.396(3) Å after thermal treatment, which was very close to the PuO_2_ obtained under hydrothermal conditions. The crystallite sizes calculated by the XRD were 24.8 ± 0.5 nm for the hydrothermally treated powder and 14.3 ± 0.6 nm for the calcined powder. In this specific case, the lower crystal size obtained by applying the thermal-decomposition method was the results of a combination of factors (we have to keep in mind that for lower hydrothermal decomposition, the typical values for the crystal size ranged from 10 to 17 nm). First, the pressure in the reactor increases compared with when a higher amount of Pu(C_2_O_4_)_2_·6H_2_O is used and, thus, the crystallites’ size increases, as demonstrated previously in the case of the UO_2_ nanopowders [16]. Moreover, the duration of the reaction is far shorter (6 h for thermal decomposition compared to 28 h for hydrothermal decomposition). Further factors that potentially exert an influence are the mechanisms of decomposition of Pu(C_2_O_4_)_2_·6H_2_O and the reaction intermediates appearing during thermal/hydrothermal decomposition [23,25]. However, as a general rule, the thermal-decomposition method gives larger particles, as discussed below.

The residual carbon content of the final product after thermal or hydrothermal decomposition was low in both cases. For the thermal decomposition in an argon atmosphere, it responded to 0.7 wt.%, which was twice as high as the value of the wet-chemistry route at 0.35 wt.% (Figure 4). In the case of dry thermal decomposition, the carbon is converted to CO/CO_2_ gases during treatment. If there is enough oxygen in the system, as is the case in an air atmosphere, more carbon can escape as CO/CO_2_. The situation is different when the powder is treated under argon, as shown by the study by Nissen et al. [29]. They found a carbon content of about 1 wt.% when Pu oxalate was heated in argon, 0.5–0.6 wt.% when it was treated in air, and only 0.15 wt.% when it was decomposed under an oxygen atmosphere [29]. A similar value was obtained by Vigier et al., who also reported 1 wt.% carbon in PuO_2_ powder prepared at 650 °C under argon [25].

The residual carbon of 0.35 wt.% for the hydrothermal decomposition of Pu(IV) oxalate observed here compares well with the carbon content of *An*O_2_ derived from the hydrothermal decomposition of Th(IV) oxalate at 250 °C for 24 h (0.3–0.8 wt.%) [21]. For the hydrothermal conversion of U(IV) oxalate at 220 °C for 24 h, a value as low as 0.01 wt.% was reported [20]. The average lattice parameter *a* was approximately the same for both samples (5.396(3) Å for the thermally treated sample and 5.397(1) Å for the sample after hydrothermal conversion). These values are in agreement with the works of Walter et al. and Bouëxière et al. [15,30].

**Figure 5:**
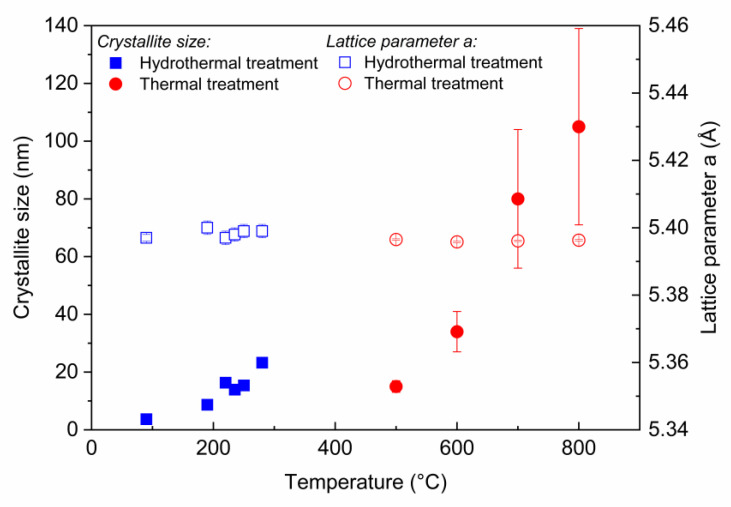
Crystallite size and lattice parameter a as a function of temperature, determined for the hydrothermally converted PuO_2_ samples (blue) and for the PuO_2_ samples (red) obtained by thermal decomposition of Pu(IV) oxalate (J.-F. Vigier, unpublished data). The data points at T = 95 °C are from Walter et al. (PuO_2_ obtained by hydrothermal conversion of Pu(IV) oxalate). Lower error bar is included in the symbols.

Finally, we compared the crystallite sizes and lattice parameters obtained by both the thermal and hydrothermal treatments relative to the literature data [15,16] and unpublished results on the thermal-decomposition process. It can be observed that the lattice parameter is essentially constant and independent of the method applied and of the reaction conditions (Figure 5). Moreover, this is very close to the lattice parameter of bulk PuO_2_ [31,32,33]. By contrast, the crystallite size is strongly affected by the method (and, hence, the temperature). The hydrothermal decomposition produced crystals from 4 to 25 nm as a function of the applied temperature and reaction time. In the case of the thermally induced process, the decomposition product was of about 15 nm at 500 °C and increased drastically to values exceeding 100 nm at an applied temperature of 800 °C.

## 4. Conclusions

Here, we presented the influence of temperature and time on the hydrothermal conversion of Pu(IV) oxalate to nanometric-size PuO_2_ with a fluorite-like structure. We also gave the first evidence of the hydrothermal conversion of Pu(III) oxalate into PuO_2_ with similar properties. The decomposition temperature can be applied to tune the crystallite size, while the duration of the treatment does not have a major influence on the obtained powder as long as the minimum duration time is respected. The crystal-lattice parameter *a* is constant and independent of the reaction conditions.

The comparison of the hydrothermal and thermal decomposition of plutonium oxalate into PuO_2_ showed positive effects, such as a lower reaction temperature and (sometimes) a shorter reaction time when using the hydrothermal technique. This could enable an alternative and cost-saving approach to obtaining spherical agglomerates of PuO_2_ nanoparticles with lower residual carbon content.

With the findings presented here, we aimed to show that the hydrothermal decomposition of plutonium oxalates under various conditions always leads to a relatively condition-independent nanometric-sized PuO_2_ with reproducible properties. These results are important as they show that the product distribution is quite independent of the reaction conditions, which could enable or facilitate larger-scale production. Therefore, the low temperature of the hydrothermal decomposition of plutonium oxalate and its stable product formation could be advantageous.

## Data Availability

Not applicable.
